# Remote Patient Monitoring Program Components and Short-Term Hypertension Control: Retrospective Cohort Study

**DOI:** 10.2196/69546

**Published:** 2026-03-24

**Authors:** Sarah LaPointe, Michael Merrill

**Affiliations:** 1 Department of Epidemiology, Rollins School of Public Health, Emory University, 1518 Clifton Rd, Atlanta, GA, 30322, United States, 1 5184867300; 2 Research Department, Brook Health, Seattle, WA, United States

**Keywords:** remote patient monitoring, RPM, hypertension control, nurse monitoring, hypertension, engagement

## Abstract

**Background:**

Remote patient monitoring (RPM) is recommended for hypertension control. However, less is known about short-term responses of hypertension to RPM and which program components are most important for hypertension control.

**Objective:**

This study aimed to evaluate the association between frequency of blood pressure monitoring, nurse monitoring, and their combination and hypertension control within 3 months.

**Methods:**

This retrospective cohort study was conducted among a convenience sample of 1464 patients with hypertension enrolled in the Brook Remote Care RPM program who sought care at any of the 68 participating primary care clinics in New York or Massachusetts, United States, between 2021 and 2023. Patients with at least 3 blood pressure measures for 70% of the weeks they were in the program were defined as having adequate readings. Brook nurses monitored patients from certain clinics, whereas clinic nurses monitored other patients. Hypertension control was defined as a weekly average blood pressure of less than 140/90 mm Hg. Generalized linear models with a binomial specification and log link were used to estimate the prevalence ratio (PR) and 95% CI of the mutually adjusted associations between adequate blood pressure readings and Brook nurse monitoring, as well as their combination with hypertension control, at 4, 8, and 12 weeks, adjusted for patient age and sex.

**Results:**

At weeks 4, 8, and 12, patients with adequate readings had 10% (PR=1.10, 95% CI 1.02-1.20), 12% (PR=1.12, 95% CI 1.04-1.20), and 15% (PR=1.15, 95% CI 1.07-1.24) higher prevalence of hypertension control than patients with lower frequencies of readings, respectively. Brook nurse monitoring was associated with 16% (PR=1.16, 95% CI 1.07-1.27), 6% (PR=1.06, 95% CI 0.99-1.15), and 8% (PR=1.08, 95% CI 1.00-1.16) higher prevalence of hypertension control at weeks 4, 8, and 12, respectively, compared to clinical staff monitoring. The combination of adequate readings plus Brook nurse monitoring was associated with 26% (PR=1.26, 95% CI 1.11-1.44), 17% (PR=1.17, 95% CI 1.05-1.31), and 27% (PR=1.27, 95% CI 1.12-1.43) higher prevalence of hypertension control at weeks 4, 8, and 12, respectively, compared to an inadequate number of blood pressure readings and not receiving Brook nurse monitoring.

**Conclusions:**

This is the first study to compare outcomes for RPM patients using clinical nurse monitoring and independent staff monitoring. This study represents a contribution to the literature on clinical outcomes for RPM patients with a focus on short-term, rather than longer-term, hypertension control, evaluation of nurse monitoring conducted by Brook Health compared to clinical staff, and the joint association of monitoring frequency and Brook nurse monitoring on hypertension control. Our study findings suggest that both frequency of monitoring and specialized nurse monitoring improve hypertension control within 3 months of program enrollment. The combination of higher monitoring frequency and Brook nurse monitoring may serve as a cost-effective approach to hypertension control in high-risk populations that overcomes the limitations of clinical care staff.

## Introduction

In the United States, the prevalence of hypertension in the adult population is 48%, and only 22% achieve hypertension control with a blood pressure of less than 140/90 mm Hg [1]. Patients with hypertension incur approximately US $2000 higher medical expenditures than their counterparts without hypertension, accounting for a total health care cost of US $130 billion [2]. While hypertension may contribute to premature mortality, patients can be treated, and progression can be prevented to achieve hypertension control.

The World Health Organization’s guidelines on the treatment of hypertension in adults recommend telemonitoring and community- or home-based self-care to improve blood pressure control as part of an integrated system, although the certainty of the evidence supporting these recommendations has been rated as low [3]. Patients with chronic conditions, including hypertension, have reported improved knowledge of their health status, more timely care, and better self-management of their conditions using remote monitoring [4]. A meta-analysis of 18 randomized controlled trials found that remote patient monitoring (RPM) lowered systolic blood pressure by 4.2 mm Hg and diastolic blood pressure by 2.4 mm Hg and increased the proportion of patients with hypertension control by 11% at 1 year of follow-up [5].

Beyond home monitoring, clinical guidelines recommend lifestyle changes and medication adherence for hypertension management and prevention [6]. Both lifestyle and pharmacological interventions have been shown to be cost-effective approaches for the reduction of blood pressure [7,8]. Thus, interest in the potential clinical benefits of joint programs that offer remote monitoring and lifestyle management for hypertension control has grown. In remote settings, nurses generally coordinate care for RPM and provide essential services for patients, including medication titration and lifestyle modification consultations [9-11].

Studies have shown that RPM programs coupled with care coordination are more effective for blood pressure reduction and hypertension control than RPM alone [12-14]. Previous studies have compared differences in average blood pressure and hypertension control between RPM and usual care groups at 3, 6, and 12 months [15,16]. However, results from a non–physician-led, entirely remote hypertension management program suggest that hypertension control is achievable at 7 weeks on average [17]. Moreover, an information and communications technology–based integrated care model showed clinically meaningful reductions in systolic blood pressure at 4 weeks [18].

A recent systematic review found that nurse-led telehealth interventions reduced blood pressure, more specifically, systolic blood pressure, based on randomized controlled trials and quasi-experimental studies [19]. Among the studies that were included in this review, few assessed the effectiveness of RPM and care coordination independently for hypertension control. Choi et al [13] found that patients who conducted remote blood pressure monitoring and received weekly virtual consultations had significantly lower systolic and diastolic blood pressure after 8 weeks than patients conducting blood pressure monitoring alone. Brennan et al [20] showed that privately insured African American individuals with hypertension who received a high-intensity, multimodal disease management program with nurse support had lower systolic blood pressure on average, had higher odds of achieving optimal blood pressure, and were more likely to record blood pressure at least once a week compared to a control group with a light-support educational program after 12 months. Hebert et al [21] compared a blood pressure monitoring intervention alone and nurse management in concert with blood pressure monitoring to usual care and found that the nurse management with blood pressure monitoring group had significantly lower systolic blood pressure at 9 months than the usual care group. No differences in systolic or diastolic blood pressure or hypertension control were observed for the blood pressure monitoring alone group at 9 or 18 months. While these studies demonstrate the effectiveness of nurse-led hypertension telehealth interventions, the specific components that affect these changes remain unclear. Moreover, all studies included in the aforementioned review by Kappes et al [19] assessed outcomes at 2 to 18 months. Therefore, there remains a gap in knowledge on the short-term independent and joint impacts of blood pressure monitoring and nurse monitoring among a diverse population of patients with hypertension.

Importantly, existing studies have explored whether nurse-led programs are effective for hypertension. There is a paucity of evidence on whether independent RPM companies with full-time nursing staff dedicated to monitoring patients offer more benefits than monitoring provided by clinical nursing staff. Given the costs of remote care [22], more research is needed to assess the short-term responses of hypertension to components of RPM and care coordination and whether RPM companies offer cost-effective programming. Therefore, the objective of this study was to examine the associations between both frequency of blood pressure readings and nurse monitoring and hypertension control at 4, 8, and 12 weeks for a population of patients with hypertension enrolled in an ongoing RPM program with nurse monitoring. We hypothesized that patients who recorded their blood pressure more frequently and who received monitoring from Brook nurses were more likely to achieve hypertension control within 3 months of program enrollment.

## Methods

### Study Design and Settings

The data used for this retrospective cohort study were drawn from a convenience sample of patients with hypertension enrolled in Brook Health’s RPM program between December 2020 and December 2023. Brook Health is a commercial digital health company that offers continuous telemonitoring programs that include a mobile app with educational content and tracking tools, telemonitoring devices, health coaching, and remote nurse monitoring and care coordination to individuals with various chronic medical conditions (eg, diabetes, hypertension, obesity, chronic obstructive pulmonary disease, and congestive heart failure) who sought care at any of the 68 (36 as of December 2023) participating primary care clinics in New York or Massachusetts, United States.

Patient eligibility for Brook RPM referral is clinic specific; however, only patients with low or no copays were enrolled in the Brook RPM program. If interested and eligible, patients were then provided with remote monitoring instruments based on their diagnoses (ie, a blood pressure cuff, scale, pulse oximeter, and/or glucose monitor) and were provided with a health coach and nurse monitor. Duration of patient monitoring, coaching, and nursing is jointly determined by health care providers and patients, but patients are in the program for 12 to 15 months on average. The independent variables in this analysis included Brook nurse monitoring compared to clinical staff monitoring and frequency of blood pressure monitoring. The dependent variable was hypertension control within 12 weeks of program initiation.

### Ethical Considerations

Approval for this secondary analysis of existing data without additional consent was provided by the University at Buffalo Institutional Review Board (00007012). Waiver of informed consent for primary and secondary data analysis was granted by the University at Buffalo Institutional Review Board as the study presents no more than minimal risk to patients. A deidentified, limited dataset was used for this study, and data access was limited to the research team to protect participant information. No compensation was provided to study participants as we used deidentified, secondary data. No identification of individual participants is possible in any images included in the manuscript or supplementary materials.

### Variables

Patients enrolled in the Brook RPM hypertension program were provided with remote cellular blood pressure cuffs (BodyTrace and Tellihealth). Patients with obesity diagnoses were also provided with remote scales (BodyTrace and Withings) to monitor weight (in kilograms) and physical activity trackers (Fitbit) to monitor physical activity (in seconds). Weight was converted to pounds, and physical activity was converted to minutes.

### Brook RPM Program Components

Brook conducted nurse monitoring for most participating clinics during the study years, whereas the remainder of the clinics conducted monitoring using their own nursing staff. Brook RPM nurse monitors and health coaches provided personalized recommendations to patients based on patient need and diagnosis. Generally, nurse monitors encouraged patients to record at least one blood pressure reading per day, although other patients were advised to record blood pressure more frequently depending on hypertension severity and presence of other comorbidities. Patients were instructed to record their blood pressure 1 to 2 hours after taking medication, before activity, or after 15 to 30 minutes of postactivity rest.

Each patient on the monitoring service received a phone call from a nurse once a month. These monthly calls were used to check patients’ status, as well as to reinforce medication compliance and adherence to device use. The monthly phone call interaction was also used to reinforce signs and symptoms for which they should call their physician or 911 to ensure that urgent and emergent care were obtained when necessary. Patients also received calls as needed based on routine readings for symptom checks and validations for any readings that were outside of the designated parameters; these calls offered opportunities for early intervention.

Patient systolic and diastolic blood pressure measured using remote blood pressure cuffs was directly transmitted to the Brook data server. The first date with a blood pressure recording was considered a patient’s activation date. We calculated baseline values of systolic and diastolic blood pressure using the average of all blood pressures recorded in the first week following the activation date. To determine whether patients adhered to blood pressure monitoring protocols, we first counted the total number of weeks with at least 3 blood pressure measurements as Brook Health patients recorded blood pressure 3 days a week on average. We calculated the proportion of weeks with at least 3 blood pressure measurements with the total number of weeks in the program as the denominator for each patient. Finally, we defined patients with at least 70% of their weeks in the program with at least 3 blood pressure measurements as patients with adequate blood pressure readings. On average, Brook Health patients submitted blood pressure readings 3 days a week for more than half of the weeks they were enrolled in the program; therefore, we selected 70% of weeks in the program to capture the patients with highest monitoring activity.

### Blood Pressure Outcomes

The primary outcome for this study was hypertension control, which we defined as an average weekly systolic blood pressure of less than 140 mm Hg and diastolic blood pressure of less than 90 mm Hg in weeks 4, 8, and 12 of the Brook RPM program.

### Covariates

**Figure 1.**
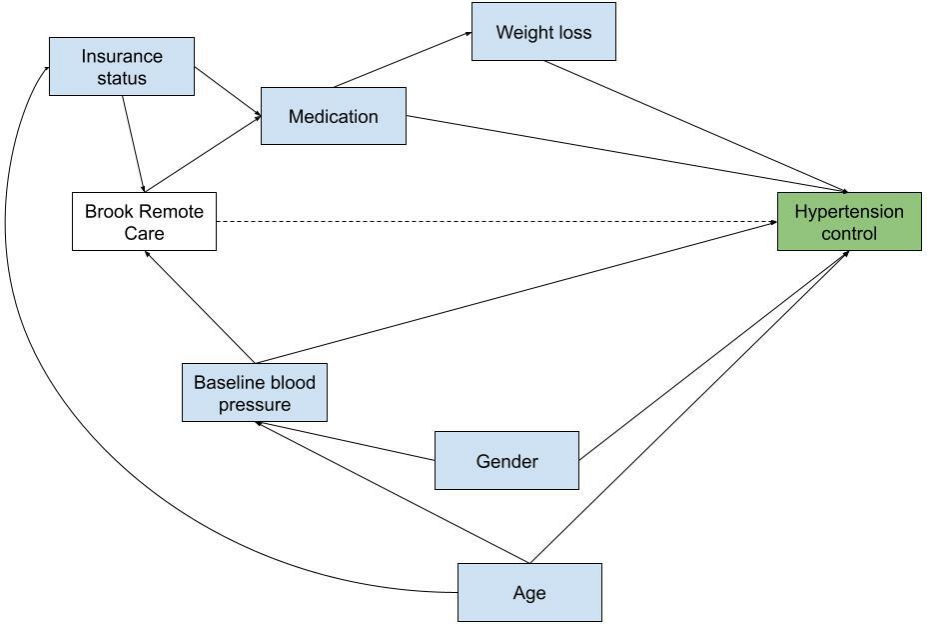
Directed acyclic graph of the association between Brook Remote Care and hypertension control.

Potential confounders in this study were identified a priori using directed acyclic graphs (Figure 1). Patient age, sex, and health insurance type were collected from patient electronic health records. Diagnoses of patient comorbidities that informed patient care and follow-up were reported as binary indicators. Baseline weight (in pounds) and physical activity (in minutes) included the first measures for each patient.

### Statistical Analysis

Patient demographic and health characteristics were presented using counts and percentages for binary variables and medians and IQRs for continuous variables. We applied chi-square tests to assess the bivariate associations between blood pressure control at 4, 8, and 12 weeks and both adequate blood pressure readings and Brook nurse monitoring. To estimate the association between both adequate blood pressure readings and Brook nurse monitoring and hypertension control, we used generalized linear models with a log link and binomial family (*epitools* package) [23] mutually adjusted for Brook nurse monitoring and adequate blood pressure readings, respectively; age; and sex. All analyses were conducted using R (version 4.3.1; R Foundation for Statistical Computing) [24]. This study was conducted in accordance with the STROBE (Strengthening the Reporting of Observational Studies in Epidemiology) reporting guidelines (Checklist 1).

### Sensitivity Analysis

To control for the potential influence of other comorbidities on these associations, we conducted an analysis among a subset of patients diagnosed with hypertension only at 4, 8, and 12 weeks using adjusted generalized linear models with a log link and binomial family.

## Results

**Figure 2.**
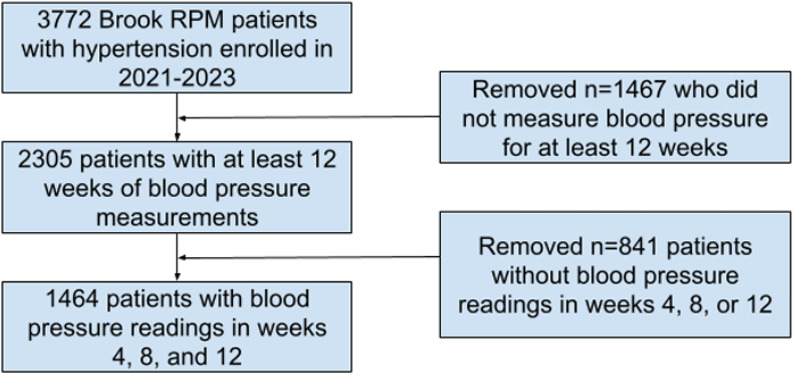
Diagram of analytic sample selection of Brook remote patient monitoring (RPM).

Our study sample included 38.8% (1464/3772) of the patients enrolled in the Brook RPM program for hypertension (Figure 2). The median age of the patients was 71 (IQR 64-78) years (Table 1). In total, 52.9% (775/1464) of the patients were female, and 67.7% (991/1464) were enrolled in the Brook RPM program in 2023. At baseline, median systolic and diastolic blood pressure were 136.8 (IQR 126.6-149.0) mm Hg and 80.8 (IQR 73.0-88.4) mm Hg, respectively. At baseline, 12.2% (179/1464) of the patients were diagnosed with diabetes, 25.1% (368/1464) were diagnosed with obesity, 1.6% (23/1464) were diagnosed with chronic obstructive pulmonary disease, and 13.6% (199/1464) were diagnosed with congestive heart failure. Patient characteristics were similar when comparing the group of patients who recorded adequate blood pressure readings and received Brook nurse monitoring to those with neither (Multimedia Appendix 1).

**Table 1.** Characteristics of patients enrolled in the Brook remote patient monitoring program for hypertension overall, by presence of adequate readings, and by receipt of Brook nurse monitoring (N=1464).

Characteristic	Overall	Adequate blood pressure readings		Brook nurse monitoring	
		No (n=606)	Yes (n=858)	No (n=528)	Yes (n=936)
Age (y), median (IQR)	71.0 (64.0-78.0)	70.0 (62.0-77.0)	72.0 (66.0-79.0)	71.0 (60.0-80.0)	72.0 (66.0-78.0)
Sex, n/N (%)					
Female	775/1464 (52.9)	339/606 (55.9)	436/858 (50.8)	272/528 (51.5)	503/936 (53.7)
Male	689/1464 (47.1)	267/606 (44.1)	422/858 (49.2)	256/528 (48.5)	433/936 (46.3)
Health insurance type, n/N (%)					
Commercial	19/435 (4.4)	13/194 (6.7)	6/241 (2.5)	13/299 (4.3)	6/136 (4.4)
Medicaid	48/435 (11.0)	28/194 (14.4)	20/241 (8.3)	47/299 (15.7)	1/136 (0.7)
Medicare	368/435 (84.6)	153/194 (78.9)	215/241 (89.2)	239/299 (79.9)	129/136 (94.9)
Participants with missing data	1029/1464 (70.3)	412/606 (68.0)	617/858 (71.9)	229/528 (43.4)	800/936 (85.5)
Activation y, n/N (%)					
2021	77/1464 (5.3)	53/606 (8.7)	24/858 (2.8)	0/528 (0.0)	77/936 (8.2)
2022	396/1464 (27.0)	184/606 (30.4)	212/858 (24.7)	209/528 (39.6)	187/936 (20.0)
2023	991/1464 (67.7)	369/606 (60.9)	622/858 (72.5)	319/528 (60.4)	672/936 (71.8)
Baseline health metrics, median (IQR)					
Systolic blood pressure (mm Hg)	136.8 (126.6-149.0)	136.4 (126.5-149.0)	137.0 (126.7-148.5)	139.2 (127.8-150.3)	135.9 (126.0-148.1)
Diastolic blood pressure (mm Hg)	80.8 (73.0-88.4)	81.8 (74.0-90.0)	80.0 (72.5-87.8)	82.7 (73.8-91.1)	79.9 (72.8-87.0)
Weight (lbs)^a^	195.2 (163.1-236.9)	200.4 (169.3-238.3)	192.9 (159.8-232.1)	182.3 (150.6-216.2)	202.3 (170.4-240.6)
Physical activity (h)^b^	0.3 (0.1-0.6)	0.3 (0.1-0.7)	0.3 (0.0-0.5)	0.4 (0.3-0.7)	0.3 (0.0-0.5)
Baseline comorbidities, n/N (%)					
Diabetes					
No	1285/1464 (87.8)	527/606 (87.0)	758/858 (88.3)	513/528 (97.2)	772/936 (82.5)
Yes	179/1464 (12.2)	79/606 (13.0)	100/858 (11.7)	15/528 (2.8)	164/936 (17.5)
Obesity					
No	1096/1464 (74.9)	457/606 (75.4)	639/858 (74.5)	491/528 (93.0)	605/936 (64.6)
Yes	368/1464 (25.1)	149/606 (24.6)	219/858 (25.5)	37/528 (7.0)	331/936 (35.4)
COPD^c^					
No	1441/1464 (98.4)	599/606 (98.8)	842/858 (98.1)	525/528 (99.4)	916/936 (97.9)
Yes	23/1464 (1.6)	7/606 (1.2)	16/858 (1.9)	3/528 (0.6)	20/936 (2.1)
Congestive heart failure					
No	1265/1464 (86.4)	522/606 (86.1)	743/858 (86.6)	414/528 (78.4)	851/936 (90.9)
Yes	199/1464 (13.6)	84/606 (13.9)	115/858 (13.4)	114/528 (21.6)	85/936 (9.1)

a Missing 774 observations.

b Missing 1188 observations.

c COPD: chronic obstructive pulmonary disease.

In unadjusted bivariate analyses, Brook RPM patients who measured their blood pressure at least 3 times a week for at least 70% of the weeks they were enrolled in the program (ie, “adequate” readings) had higher proportions of hypertension control at 4 weeks (557/858, 64.9% vs 358/606, 59.1%; *P*=.03), 8 weeks (613/858, 71.4% vs 388/606, 64.0%; *P*=.003), and 12 weeks (622/858, 72.5% vs 379/606, 62.5%; *P*<.001) than patients who did not submit adequate readings (Table 2). A higher proportion of patients receiving Brook nurse monitoring achieved hypertension control at week 4 (616/936, 65.8% vs 299/528, 56.6%; *P*<.001), week 8 (653/936, 69.8% vs 348/528, 65.9%; *P*=.14), and week 12 (659/936, 70.4% vs 342/528, 64.8%; *P*=.03) than patients receiving nurse monitoring from nursing staff at their primary care clinics. Among the group of patients who recorded adequate blood pressure readings and received Brook nurse monitoring, 68.8% (379/551), 73.5% (405/551), and 74.2% (409/551) achieved hypertension control at 4, 8, and 12 weeks, respectively (Multimedia Appendix 2).

In multivariable analyses, patients with adequate blood pressure readings had higher prevalence of hypertension control at week 4 (prevalence ratio [PR]=1.10, 95% CI 1.02-1.20), week 8 (PR=1.12, 95% CI 1.04-1.20), and week 12 (PR=1.15, 95% CI 1.07-1.24) than those who did not have adequate readings (Table 3). In models mutually adjusted for adequate readings, patients who received Brook nurse monitoring had higher prevalence of hypertension control at week 4 (PR=1.16, 95% CI 1.07-1.27), week 8 (PR=1.06, 95% CI 0.99-1.15), and week 12 (PR=1.08, 95% CI 1.00-1.16) than patients monitored by staff at primary care clinics. Patients who recorded adequate blood pressure readings and received Brook nurse monitoring (772/1464, 52.7%) had higher prevalence of hypertension control at week 4 (PR=1.26, 95% CI 1.11-1.46), week 8 (PR=1.17, 95% CI 1.05-1.31), and week 12 (PR=1.27, 95% CI 1.12-1.43) than patients who recorded blood pressure less frequently and who received remote monitoring from clinical staff. These associations among adequate readings, nurse monitoring, and blood pressure control were generally similar, though slightly higher in magnitude, in the sensitivity analyses (Multimedia Appendix 3).

**Table 2.** Bivariate associations between both adequate blood pressure readings and Brook nurse monitoring and hypertension control at 4, 8, and 12 weeks (N=1464).

	Adequate readings			Brook nurse monitoring		
	No (n=606), n (%)	Yes (n=858), n (%)	*P* value^a^	No (n=528), n (%)	Yes (n=936), n (%)	*P* value^a^
Hypertension control at 4 wk			.03			<.001
No	248 (40.9)	301 (35.1)		229 (43.4)	320 (34.2)	
Yes	358 (59.1)	557 (64.9)		299 (56.6)	616 (65.8)	
Hypertension control at 8 wk			.003			.14
No	218 (36.0)	245 (28.6)		180 (34.1)	283 (30.2)	
Yes	388 (64.0)	613 (71.4)		348 (65.9)	653 (69.8)	
Hypertension control at 12 wk			<.001			.03
No	227 (37.5)	236 (27.5)		186 (35.2)	277 (29.6)	
Yes	379 (62.5)	622 (72.5)		342 (64.8)	659 (70.4)	

a Pearson chi-square test.

**Table 3.** Associations between Brook Remote Care program components and hypertension control at 4, 8, and 12 weeks in the program. Models were adjusted for patient age and sex.

	Hypertension control prevalence ratio (95% CI)		
	4 wk	8 wk	12 wk
Adequate readings (N=1464)			
No	Reference	Reference	Reference
Yes	1.10 (1.02-1.20)	1.12 (1.04-1.20)	1.15 (1.07-1.24)
Brook nurse monitoring (N=1464)			
No	Reference	Reference	Reference
Yes	1.16 (1.07-1.27)	1.06 (0.99-1.15)	1.08 (1.00-1.16)
Adequate readings and Brook nurse monitoring combined (n=772)			
No	Reference	Reference	Reference
Yes	1.26 (1.11-1.44)	1.17 (1.05-1.31)	1.27 (1.12-1.43)

## Discussion

### Principal Findings

This longitudinal retrospective cohort study among a convenience sample of patients at any of the 68 participating primary care clinics across New York and Massachusetts, United States, enrolled in the Brook RPM program for hypertension examined the associations between 2 Brook RPM program components—Brook nurse monitoring relative to clinical nurse monitoring and frequency of blood pressure readings—and hypertension control at 4, 8, and 12 weeks. Patients who recorded blood pressure more than 70% of their time in the program had higher prevalence of hypertension control than those who did not. Brook nurse monitoring was associated with greater prevalence of hypertension control than clinical nursing staff monitoring. The group of patients with high frequency of blood pressure readings and who received Brook nurse monitoring had the highest prevalence of hypertension control compared to all other groups.

Evaluation of the frequency of blood pressure readings recorded by patients is important to guide clinical recommendations for RPM. Frazier et al [25] observed that mean arterial pressure reductions were greatest for patients with higher frequencies of measurements per month over the course of a 6-month RPM program. Despite the Centers for Medicare and Medicaid Services stipulation that Current Procedural Terminology codes for initial equipment setup and monthly data collection are billed only for patients who record at least 16 measurements per month [26], there is no evidence documenting the clinical relevance of these suggested monitoring frequencies. Therefore, more studies evaluating the clinical implications of monitoring intensity are needed.

Team-based care has been recommended as a cost-effective approach for patients’ hypertension control and management [27]. Our study observed that, compared to nurse monitoring provided by clinical staff, Brook nurse monitoring was consistently associated with higher hypertension control among patients, highlighting that the quality of nurse interactions may be more impactful for hypertension control than simply having a nurse monitor present. A pragmatic observational cohort study among Medicare patients with uncontrolled hypertension who sought care at primary care clinics in Chicago, Illinois (age range 65-85 years), and the general hypertension population found that RPM plus care coordination provided by nurses showed clinically meaningful improvements in hypertension control at 3 and 6 months compared to usual care and RPM alone [12]. Additionally, a cluster randomized trial of the HyperLink RPM program that provided pharmacist-led care coordination to patients attending primary care in Minnesota showed that the telemonitoring intervention had 25% to 30% higher absolute hypertension control at 6 and 12 months than usual care [28]. An experimental longitudinal study among African American individuals with uncontrolled hypertension found that a 12-month nurse-managed telemonitoring program that provided RPM and nurse calls to patients led to statistically and clinically significant reductions in systolic blood pressure compared to usual care, with the greatest reductions observed in the first 3 months of the program [9]. Moreover, in a mediation analysis, secure messaging with a pharmacist accounted for 96% of the effects of an RPM program on hypertension control after 12 months [29].

A unique contribution of our Brook RPM study is the short-term hypertension control response examined at 4, 8, and 12 weeks in relation to program components. Most studies have evaluated digital health interventions in relation to hypertension control and blood pressure outcomes at longer periods, such as 6 and 12 months of program participation [19,30]. However, there is evidence that RPM shows promise for improving blood pressure outcomes at earlier time points [17,18,31]. Naqvi et al [31] observed an 18.4 mm Hg reduction in systolic blood pressure among those using RPM after 3 months compared to controls. A non–physician-led hypertension management program that included a Bluetooth-enabled home blood pressure device was shown to lead to hypertension control for 91% of patients after approximately 7 weeks of consistent measurement [17]. A study among 20 patients with difficult-to-control hypertension enrolled in an information and communications technology–based program that included telemonitoring and weekly access to nurses showed that systolic blood pressure measured at home decreased by 15 mm Hg on average after 4 weeks [18]. In concert with these study findings, our study showed improved blood pressure control among patients who recorded blood pressure more frequently and who received Brook nurse monitoring at 4, 8, and 12 weeks.

### Limitations

Our study findings have limited generalizability as Brook RPM patients are older, reside in western New York or eastern Massachusetts, and are generally enrolled in Medicare health insurance plans that cover RPM costs with low to no patient cost sharing. Patients who received Brook nurse monitoring were identified based on primary care clinics, as Brook nurses conduct monitoring for patients at certain clinics. This definition masks the variability in individual patient experiences as monitoring protocols are tailored to patient needs. Moreover, the level of monitoring provided by clinical staff and the differences between staff within the same clinic as well as between clinics are unclear as clinical monitoring protocols are not made available to Brook Health. Additionally, most Brook patients were encouraged to monitor their blood pressure at least once a day. However, we used a more data-driven estimate of monitoring intensity to define adequate blood pressure readings in this study (ie, 70% of weeks in the program with at least 3 days of blood pressure readings) as most patients did not consistently record their blood pressure every day for all weeks in the program. The use of a complete-case analysis may introduce bias into our study, and we encourage cautious interpretation of study findings. Additionally, uncontrolled confounding may bias our study findings as we lacked information on important demographic and clinical characteristics that likely confound these associations, and health insurance information was incomplete and not accounted for in our analysis due to a high degree of missingness. For instance, baseline blood pressure used to determine eligibility for the Brook Health program was not available for this study as it was based on clinical medical records, and patient medication adherence was only available through nurse notes, which were not complete. Therefore, our study may be subject to residual confounding that may bias our results. However, it is more likely that these variables do not act as true confounders but rather serve as mediators between the program’s nurse monitoring component and hypertension control.

### Conclusions

This is the first study to compare short-term hypertension control outcomes for RPM patients using clinical nurse monitoring and independent RPM staff monitoring. Our study findings suggest that both frequency of monitoring and specialized nurse monitoring improve hypertension control within 3 months of program enrollment. The combination of higher monitoring frequency and Brook nurse monitoring may represent a cost-effective approach to improving hypertension control in high-risk populations by addressing limitations in clinical care staffing. Professional RPM companies with full-time staff may augment the benefits of RPM provided by clinical care providers who have other responsibilities. Future studies should further evaluate the impact of individual and collective program components on health outcomes and assess these potential impacts at various time points of program participation.

## Supplementary material

10.2196/69546 Multimedia Appendix 1 Characteristics of patients enrolled in the Brook remote patient monitoring program for hypertension overall, by presence of adequate readings, and by receipt of Brook nurse monitoring.

10.2196/69546 Multimedia Appendix 2 Bivariate associations between adequate blood pressure readings and Brook nurse monitoring combined and hypertension control at 4, 8, and 12 weeks (n=772).

10.2196/69546 Multimedia Appendix 3 Associations between Brook Remote Care program components and hypertension control at 4, 8, and 12 weeks in the program among patients diagnosed with hypertension only (n=864).

10.2196/69546 Checklist 1 STROBE checklist.

## Abbreviations

PR: prevalence ratio
RPM: remote patient monitoring
STROBE: Strengthening the Reporting of Observational Studies in Epidemiology

## References

[1] (2023). Hypertension cascade: hypertension prevalence, treatment and control estimates among US adults aged 18 years and older applying the criteria from the American College of Cardiology and American Heart Association’s 2017 hypertension guideline—NHANES 2017–2020. https://millionhearts.hhs.gov/data-reports/hypertension-prevalence.html.

[2] Kirkland EB, Heincelman M, Bishu KG (2018). Trends in healthcare expenditures among US adults with hypertension: national estimates, 2003-2014. J Am Heart Assoc.

[3] (2021). Guideline for the pharmacological treatment of hypertension in adults. World Health Organization.

[4] Walker RC, Tong A, Howard K, Palmer SC (2019). Patient expectations and experiences of remote monitoring for chronic diseases: systematic review and thematic synthesis of qualitative studies. Int J Med Inform.

[5] Cappuccio FP, Kerry SM, Forbes L, Donald A (2004). Blood pressure control by home monitoring: meta-analysis of randomised trials. BMJ.

[6] Whelton PK, Carey RM, Aronow WS (2018). 2017 ACC/AHA/AAPA/ABC/ACPM/AGS/APhA/ASH/ASPC/NMA/PCNA guideline for the prevention, detection, evaluation, and management of high blood pressure in adults: a report of the American College of Cardiology/American Heart Association Task Force on Clinical Practice Guidelines. Hypertension.

[7] Rubinstein A, Colantonio L, Bardach A (2010). Estimation of the burden of cardiovascular disease attributable to modifiable risk factors and cost-effectiveness analysis of preventative interventions to reduce this burden in Argentina. BMC Public Health.

[8] Neter JE, Stam BE, Kok FJ, Grobbee DE, Geleijnse JM (2003). Influence of weight reduction on blood pressure: a meta-analysis of randomized controlled trials. Hypertension.

[9] Artinian NT, Flack JM, Nordstrom CK (2007). Effects of nurse-managed telemonitoring on blood pressure at 12-month follow-up among urban African Americans. Nurs Res.

[10] Bosworth HB, Olsen MK, McCant F (2007). Hypertension Intervention Nurse Telemedicine Study (HINTS): testing a multifactorial tailored behavioral/educational and a medication management intervention for blood pressure control. Am Heart J.

[11] Laurant M, Reeves D, Hermens R, Braspenning J, Grol R, Sibbald B (2005). Substitution of doctors by nurses in primary care. Cochrane Database Syst Rev.

[12] Persell SD, Petito LC, Anthony L (2023). Prospective cohort study of remote patient monitoring with and without care coordination for hypertension in primary care. Appl Clin Inform.

[13] Choi WS, Kim NS, Kim AY, Woo HS (2021). Nurse-coordinated blood pressure telemonitoring for urban hypertensive patients: a systematic review and meta-analysis. Int J Environ Res Public Health.

[14] Bosworth HB, Olsen MK, Grubber JM (2009). Two self-management interventions to improve hypertension control: a randomized trial. Ann Intern Med.

[15] McManus RJ, Mant J, Bray EP (2010). Telemonitoring and self-management in the control of hypertension (TASMINH2): a randomised controlled trial. Lancet.

[16] Omboni S (2019). Connected health in hypertension management. Front Cardiovasc Med.

[17] Fisher ND, Fera LE, Dunning JR (2019). Development of an entirely remote, non-physician led hypertension management program. Clin Cardiol.

[18] Visco V, Finelli R, Pascale AV (2018). Difficult-to-control hypertension: identification of clinical predictors and use of ICT-based integrated care to facilitate blood pressure control. J Hum Hypertens.

[19] Kappes M, Espinoza P, Jara V, Hall A (2023). Nurse-led telehealth intervention effectiveness on reducing hypertension: a systematic review. BMC Nurs.

[20] Brennan T, Spettell C, Villagra V (2010). Disease management to promote blood pressure control among African Americans. Popul Health Manag.

[21] Hebert PL, Sisk JE, Tuzzio L (2012). Nurse-led disease management for hypertension control in a diverse urban community: a randomized trial. J Gen Intern Med.

[22] American Diabetes Association (2018). Economic costs of diabetes in the U.S. in 2017. Diabetes Care.

[23] Aragon TJ, Fay MP, Wollschlaeger D, Omidpanah A (2020). epitools: epidemiology tools. The Comprehensive R Archive Network.

[24] R Core Team (2025). R: a language and environment for statistical computing. The Comprehensive R Archive Network.

[25] Frazier WD, Beins M, DaVanzo J, Heath S, Dobson A (2023). Six months of remote patient monitoring is associated with blood pressure reduction in hypertensive patients: an uncontrolled observational study. Telemed J E Health.

[26] Telehealth and remote patient monitoring. Telehealth.HHS.gov.

[27] Jacob V, Reynolds JA, Chattopadhyay SK (2023). Economics of team-based care for blood pressure control: updated community guide systematic review. Am J Prev Med.

[28] Margolis KL, Asche SE, Bergdall AR (2013). Effect of home blood pressure telemonitoring and pharmacist management on blood pressure control: a cluster randomized clinical trial. JAMA.

[29] Ralston JD, Cook AJ, Anderson ML (2014). Home blood pressure monitoring, secure electronic messaging and medication intensification for improving hypertension control: a mediation analysis. Appl Clin Inform.

[30] Katz ME, Mszar R, Grimshaw AA (2024). Digital health interventions for hypertension management in US populations experiencing health disparities: a systematic review and meta-analysis. JAMA Netw Open.

[31] Naqvi IA, Strobino K, Kuen Cheung Y (2022). Telehealth after stroke care pilot randomized trial of home blood pressure telemonitoring in an underserved setting. Stroke.

