# Supplementary material for: Remote Patient Monitoring Program Components and Short-Term Hypertension Control: Retrospective Cohort Study
Source: JMIR Mhealth Uhealth. 2026 Mar 24;14:e69546. doi: 10.2196/69546 (PMC13011998; doi:10.2196/69546)
Supplement: Multimedia Appendix 1 [file mhealth-v14-e69546-s001.pdf]

Multimedia Appendix 1. Characteristics of patients enrolled in Brook RPM for hypertension overall, by adequate readings and brook nurse monitoring

	Median (25th, 75th percentile) or n/N (%)	
	Adequate Readings and Brook Nurse Monitoring	
	No	Yes
**Number of patients**	221	551
Characteristic		
Age (years)	69.0 (58.0, 80.0)	73.0 (67.0, 79.0)
Gender		
Female	118 / 221 (53%)	282 / 551 (51%)
Male	103 / 221 (47%)	269 / 551 (49%)
Health insurance type		
Commercial	9 / 138 (6.5%)	2 / 80 (2.5%)
Medicaid	27 / 138 (20%)	0 / 80 (0%)
Medicare	102 / 138 (74%)	78 / 80 (98%)
Missing	83	471
Year of activation		
2021	0 / 221 (0%)	24 / 551 (4.4%)
2022	96 / 221 (43%)	99 / 551 (18%)
2023	125 / 221 (57%)	428 / 551 (78%)
**Baseline health metrics**		
Systolic blood pressure (mm Hg)	136.4 (126.2, 149.7)	135.0 (125.0, 147.0)
Diastolic blood pressure (mm Hg)	82.5 (73.7, 91.7)	78.8 (71.7, 86.0)
Weight (lbs)[a]	182.5 (148.8, 211.2)	197.3 (162.9, 238.5)
Physical activity (hrs)[b]	0.4 (0.2, 0.6)	0.3 (0.0, 0.5)
**Baseline comorbidities**		
Diabetes		
No	209 / 221 (95%)	454 / 551 (82%)
Yes	12 / 221 (5.4%)	97 / 551 (18%)
Obesity		
No	206 / 221 (93%)	354 / 551 (64%)
Yes	15 / 221 (6.8%)	197 / 551 (36%)
COPD		
No	219 / 221 (99%)	536 / 551 (97%)
Yes	2 / 221 (0.9%)	15 / 551 (2.7%)
Congestive heart failure		
No	166 / 221 (75%)	495 / 551 (90%)
Yes	55 / 221 (25%)	56 / 551 (10%)

COPD: Chronic obstructive pulmonary disease

[a]Missing 391 observations

[b]Missing 630 observations
