# Supplementary material for: Remote Patient Monitoring Program Components and Short-Term Hypertension Control: Retrospective Cohort Study
Source: JMIR Mhealth Uhealth. 2026 Mar 24;14:e69546. doi: 10.2196/69546 (PMC13011998; doi:10.2196/69546)
Supplement: Multimedia Appendix 2 [file mhealth-v14-e69546-s002.pdf]

Multimedia Appendix 2. Bivariate Associations of Adequate Blood Pressure Readings and Brook Nurse Monitoring Combined with Hypertension Control at 4, 8, and 12 Weeks (n=772)

	n/N (%)		p-value[1]
**Hypertension control (4 weeks)**			<.001
**No**	100 / 221 (45%)	172 / 551 (31%)	
**Yes**	121 / 221 (55%)	379 / 551 (69%)	
**Hypertension control (8 weeks)**			.007
**No**	81 / 221 (37%)	146 / 551 (26%)	
**Yes**	140 / 221 (63%)	405 / 551 (74%)	
**Hypertension control (12 weeks)**			<.001
**No**	92 / 221 (42%)	142 / 551 (26%)	
**Yes**	129 / 221 (58%)	409 / 551 (74%)	

[1] Pearson’s Chi-square test
