# Supplementary material for: Remote Patient Monitoring Program Components and Short-Term Hypertension Control: Retrospective Cohort Study
Source: JMIR Mhealth Uhealth. 2026 Mar 24;14:e69546. doi: 10.2196/69546 (PMC13011998; doi:10.2196/69546)
Supplement: Multimedia Appendix 3 [file mhealth-v14-e69546-s003.pdf]

Multimedia Appendix 3. Associations between Brook Remote Care program components and hypertension control at 4, 8, and 12 weeks in the program among patients diagnosed with hypertension only (n=864).

		**Hypertension Control Prevalence Ratios and 95% Confidence Intervals**		
	**N**	**Week 4**	**Week 8**	**Week 12**
**Adequate Readings**	**864**			
**No**		Ref	Ref	Ref
**Yes**		1.17 (1.05, 1.32)	1.18 (1.06, 1.30)	1.22 (1.10, 1.35)
**Brook Nurse Monitoring**	**864**			
**No**		Ref	Ref	Ref
**Yes**		1.30 (01.15, 1.47)	1.12 (1.01, 1.26)	1.11 (1.01, 1.23)
**Adequate Readings and Brook Nurse Monitoring Combined**	416			
**No**		Ref	Ref	Ref
**Yes**		1.40 (1.17, 1.68)	1.29 (1.11, 1.50)	1.42 (1.19, 1.69)

Models were adjusted for patient age and sex.
